# The Future of Synbiotics: Rational Formulation and Design

**DOI:** 10.3389/fmicb.2022.919725

**Published:** 2022-07-22

**Authors:** David F. Gomez Quintero, Car Reen Kok, Robert Hutkins

**Affiliations:** ^1^Department of Food Science and Technology, University of Nebraska-Lincoln, Lincoln, NE, United States; ^2^Nebraska Food for Health Center, University of Nebraska-Lincoln, Lincoln, NE, United States; ^3^Complex Biosystems, University of Nebraska-Lincoln, Lincoln, NE, United States

**Keywords:** probiotic, prebiotic, synbiotic, complementary, synergistic

## Abstract

Synbiotics, mixtures of live microbes and substrates selectively utilized by host organisms, are of considerable interest due to their ability to improve gastrointestinal health. However, formulating synbiotics remains challenging, due in part, to the absence of rational strategies to assess these products for synbiotic activities prior to clinical trials. Currently, synbiotics are formulated as either complementary or synergistic. Complementary synbiotics are made by combining probiotics and prebiotics, with each component acting independently and with the combination shown to provide a clinical health benefit. Most commercial synbiotics as well as those used in clinical trials have been of the complementary type. In contrast, synergistic synbiotics require that the added microbe is specifically stimulated or it’s persistence or activity are enhanced by the cognate substrate. Although several innovative examples have been described in the past few years based on this principle, in practice, relatively few synbiotic studies have tested for synergism. In this review, selected recent examples of complementary and synergistic synbiotics and the rationale for their formulation will be described. In addition, pre-clinical experimental approaches for identifying combinations that provide a basis for satisfying the requirements for synergism will be discussed.

## Introduction

Research over the past two decades has demonstrated that the gut microbiome has a profound influence on human and animal health (Fan and Pedersen, 2020). When the gut microbiome is disrupted by antibiotics, diet, stress, or other factors, a dysbiotic state—an imbalance or adverse departure from a microbiome of a normal healthy control may occur (Kriss et al., 2018). Such a condition may lead to the onset of a range of complex diseases and disorders (Proffitt et al., 2020; Khor et al., 2021). Thus, the specific composition of the microbiome and interactions of the microbiome with host cells in the gastrointestinal (GI) tract are now actively being studied (Kinross et al., 2011; Wu et al., 2021). Moreover, since gut microbes are responsible for digestion, carbohydrate metabolism, energy production, protection against pathogens, and immunomodulation (Barengolts, 2016), there is considerable potential in modifying the gut microbiota to enhance host health. One way of doing this is *via* consumption of probiotics and prebiotics (Krumbeck et al., 2018b).

## Synbiotic Challenges

Despite decades of research on probiotics and prebiotics, it remains challenging to obtain consistent positive clinical outcomes with these products. Certainly, this is partly due to the wide assortment of probiotic species and strains, the diversity in prebiotic structures, varying doses of probiotics and prebiotics, and the specific target outcomes that are defined in these studies. But another important consideration are the ecological constraints that influence the ability of probiotic microbes or prebiotic substrates to effect changes in the GI tract. After all, probiotics are often out-numbered a million-to-one by the commensal microbiota. For many probiotic products, the microbes are not of human origin and lack the necessary traits to compete and persist in the GI tract (Walter et al., 2018). In particular, the ability to capture and consume non-digestible carbohydrates and other specialized resources is a major factor that determines if a given microbe can occupy a niche, even transiently in the GI environment.

The effectiveness of prebiotics alone is also subject to similar personalized constraints. For example, for prebiotics to serve as selective substrates, relevant microbes capable of consuming those substrates and converting them into beneficial products or other outputs to the host, must be present in an individual’s microbiome. It is also possible that substrates that lack sufficient structural or chemical specificity instead promote non-targeted enrichment of members of the gut microbiome, resulting in a lack of beneficial effects on the host (Hamaker and Tuncil, 2014). Collectively, these ecological considerations, in addition to immunological, physiological, and other host factors, contribute to the non-responder phenotype commonly observed across different studies and study populations (Arnold et al., 2021; Ojima et al., 2022). Thus, one of the advantages of the synbiotic concept is the possibility to circumvent these ecological limitations by providing the microbe along with a substrate that supports the growth of that microbe (Swanson et al., 2020).

## Consensus Definitions

Although numerous groups have suggested definitions for these terms, arguably the most authoritative definitions are those proposed by consensus panels organized by the International Scientific Association for Probiotics and Prebiotics (ISAPP). Accordingly, probiotics were defined as “live microorganisms which when administered in adequate amounts confer a health benefit on the host” (Hill et al., 2014), and a prebiotic was defined as a “substrate that is selectively utilized by host microorganisms conferring a health benefit” (Gibson et al., 2017).

More recently, another ISAPP consensus panel defined a synbiotic as “a mixture comprising live microorganisms and substrate(s) selectively utilized by host microorganisms that confers a health benefit on the host” (Swanson et al., 2020). Interestingly, synbiotics had first been described more than 25 years ago in the paper that first introduced the prebiotic concept (Gibson and Roberfroid, 1995). Later, Kolida and Gibson (2011) distinguished between two types of synbiotics, complementary and synergistic. The former consists of a probiotic and a prebiotic, both acting independently, whereas for the latter, the prebiotic is chosen to specifically stimulate the selected probiotic.

In the synbiotic consensus review, the main features of the complementary type were retained (Swanson et al., 2020). They clarified that complementary synbiotics “are not designed so that its components function cooperatively.” Although each component of a complementary synbiotic should, by definition, independently provide a health benefit, they stated that the combined synbiotic should still be clinically demonstrated to be beneficial to the host. In contrast, for synergistic synbiotics, the panel proposed that neither a probiotic nor a prebiotic were required components. Rather, they referred to the two constituents as “substrates” and “microorganisms.” However, they did specify that the substrate should be selectively utilized by the co-administrated microorganism and that the latter is the main target of enrichment (Swanson et al., 2020). Furthermore, an important distinction between these two types is that synergistic synbiotics must provide a benefit greater than either the substrate or microbe treatments.

## Synbiotics in the Marketplace

Synbiotics (or at least products labeled as synbiotics) have received considerable consumer attention in recent years despite not being very common in either the supplement or food marketplace even a decade ago (Cielecka-Piontek et al., 2020; Li et al., 2020; Chaturvedi et al., 2021; Voss et al., 2021). Indeed, in a recent survey using data obtained from the National Health and Nutrition Examination Survey (NHANES), more than 1% of U.S. adults and children reported consumption of non-food (e.g., supplements) synbiotics, compared to 5 and 2%, respectively, for probiotics and prebiotics (O’Connor et al., 2021). Older adults (>60 years of age) in particular, were more frequent consumers of these products, with synbiotic use near 2% among this group. The authors considered these as relatively high prevalence values for non-vitamin or non-mineral dietary supplements. Likewise, food applications for synbiotics have been predicted to increase globally across both dairy and non-dairy and fermented and non-fermented categories (Mishra et al., 2021; Cosme et al., 2022). Collectively, sales of synbiotic-containing foods and supplements are nearing $1B (Cosme et al., 2022).

## General Criteria for Synbiotics

Given that definitions and characteristics of probiotics and prebiotics are now well established, satisfying the relevant criteria for each of these categories should be a straight-forward process (Binda et al., 2020; Scott et al., 2020; Cunningham et al., 2021). However, systematic approaches for formulating synbiotics that are in accordance with the ISAPP definition are lacking. Furthermore, for many of the clinical studies described in the literature, the scientific basis for combining a given probiotic with a specific prebiotic is either vague or simply not stated. In general, when a justification is given, combinations are chosen based either on the independent properties of each component or on the suggested ability of the prebiotic substrate to support growth of a known or putative probiotic microbe (Furrie et al., 2005; Scorletti et al., 2018).

To clarify these issues, one of the main goals of the ISAPP consensus panel on synbiotics was to provide guidelines and criteria for formulating both complementary and synergistic synbiotics with an emphasis placed on the need to demonstrate a health benefit through a well-designed clinical study. Thus, the ISAPP guidelines also include criteria for ensuring sufficient statistical power, appropriate blinding and randomization, and reporting of inclusion and exclusion criteria among many others.

Beyond the specific recommendations of the ISAPP report, additional criteria may also be relevant for designing studies to test synbiotics. For example, investigators should clearly state the rationale for the synbiotic pairing and if the synbiotic being tested is of the synergistic or complementary type. Accordingly, the controls must be appropriate for the study design, especially for synergistic synbiotics, such that they account for effects exhibited by either of the two individual components. Ultimately, the study design for complementary synbiotics is far easier to satisfy and test for than synergistic synbiotics.

## Formulating and Testing Complementary Synbiotics

For complementary synbiotics, the choice for each component depends on the targeted clinical endpoint and the capacity of those components to achieve that endpoint. Because the individual probiotic and prebiotic components must, by definition, each confer a health benefit, it would be expected that the combined product would also do so. Nonetheless, the synbiotic must be shown to have a health benefit in an appropriately designed, randomized controlled study (RCT). It is possible, for example, that the prebiotic is consumed by a commensal gut microbe that out-competes or inhibits the growth or biological activity of the administered probiotic, resulting in a null effect. Indeed, in one recent study, a probiotic improved constipation symptoms, but the synbiotic reduced that effect (Ito et al., 2022). Also, if a synbiotic was formulated such that the dose of one or both components were below the effective range, a clinical trial would also be warranted. Based on stated doses on the labels, many commercial synbiotic products as well as those used in clinical studies apparently contain prebiotics doses well below that which would be expected to confer a health benefit (Ghavami et al., 2021; Quero et al., 2021; Ito et al., 2022).

For many reasons—convenience, ease of formulation, and simple experimental design, nearly all synbiotics used in clinical trials have been of the complementary type. As reviewed previously (Swanson et al., 2020), many have been reported to provide important health benefits on the tested population. More recent RCTs have also been reported (Table 1), again with some showing positive outcomes, and others not.

**TABLE 1:** Summary of recent (since 2020) synbiotic clinical trials.

Microbe/day	Substrate/day	Subjects	Controls		Primary clinical outcome	References
					
			Microbe only	Substrate only		
*Bifidobacterium infantis* EVC001, 8 × 10^9^ CFU	HMO, 1.6 g	Infants with severe acute malnutrition	Yes	No	Promoted weight gain and reduced inflammation markers	Barratt et al., 2022
*Lactiplantibacillus plantarum* PBS067, *Lactobacillus acidophilus* PBS066 and *Limosilactobacillus reuteri* PBS072 2 × 10^9^ CFU each	Inulin/FOS (1:1)	Elderly patients with metabolic syndrome	No	No	Reduced MetS symptoms and cardiovascular risk factors	Cicero et al., 2021
*Lactobacillus, Bifidobacterium, and Enterococcus* (about 2.8 × 10^9^ CFU)	Plant fiber mixture, 60 g	Adult patients with mental disorders	Yes	Yes	Reduced antipsychotic-induced weight gain	Huang et al., 2022
*Bifidobacterium longum* NT	GOS, 1 g	Adults with constipation	Yes	No	Synbiotic attenuated the positive effect of the probiotic on constipation	Ito et al., 2022
*Lacticaseibacillus paracasei* strain Shirota and *Bifidobacterium breve*, each 3 × 10^8^ CFU	GOS, 7.5 g	Obese adults with T2D	No	No	No change in interleukin-6	Kanazawa et al., 2021
*Lactobacillus acidophilus, Bifidobacterium bifidum, Bifidobacterium lactis*, and *Bifidobacterium longum* (1.5 × 10^9^ CFU for each)	Inulin <6 g/day	Adults with prediabetes	Yes	No	No significant change in the gut microbiome	Kassaian et al., 2020
*Bifidobacterium animalis* subsp. lactis Vesalius 002, 10 × 10^9^ CFU	FOS, 9.9 g	Middle-aged adults	No	No	Reduction in days with abdominal discomfort	Neyrinck et al., 2021
*Bifidobacterium breve* M-16V 1 × 10^4^ or 1 × 10^6^ CFU	GOS/FOS (9:1), 6.5 g	Infants aged from 6 to 19 weeks	No	No	Increase of bifidobacteria and decrease of *Clostridioides difficile* in the synbiotic groups	Phavichitr et al., 2021
Nine strains, 10^9^ CFU	FOS, 1.43 g	Colicky infants	No	No	Higher responder rate	Piątek et al., 2021
*Bifidobacterium animalis* subspecies *lactis* BB-12 10^10^ CFU	FOS, 8 g	Patients with non-alcoholic fatty liver disease	No	No	No effect on markers of liver disease	Scorletti et al., 2020
*Lactobacillus acidophilus*, *Bifidobacterium lactis*, *Bifidobacterium longum*, and *Bifidobacterium bifidum* 15 × 10^9^ CFU	GOS, 2.75 g/day	Obese or overweight adults	No	No	Change in body composition or weight loss	Sergeev et al., 2020
Five strains, 10^10^ CFU	FOS, 1.89 g	Adult IBS patients	No	No	Improved IBS symptoms	Skrzydło-Radomańska et al., 2020
*Lactobacillus acidophilus* LA-5, 2⋅1 × 10^8^ CFU and *Bifidobacterium animalis* subspecies *lactis* BB-12, 2⋅7 × 10^9^ CFU	Inulin, 2.3 g	Military personnel, 18–22 years of age	No	No	Decreased tenseness and sleepiness	Valle et al., 2021

Perhaps the most successful synbiotic trial reported in the literature was for a complementary synbiotic to reduce sepsis in a population of infants (Panigrahi et al., 2017). This was a large RCT (more than 2,000 participants per arm) using an oral synbiotic containing *Lactiplantibacillus plantarum* ATCC 202195 (10^9^ CFU/day) and fructooligosaccharides (FOS) as the intervention. Significant reductions in sepsis and mortality were observed in the synbiotic treatment group. Although the strain had been selected based on its ability to colonize the infant gut and block adherence and translocation of Gram-negative bacteria, they did not assess for the presence and persistence of the strain in fecal samples. Thus, it was not possible to determine which component—the strain or the prebiotic, was responsible for the health outcome. Similarly, in another infant study, *Bifidobacterium breve* M-16V was combined with GOS and FOS (Phavichitr et al., 2021). Although the synbiotic enhanced abundance of the target organism and increased acetate and lactate, the study design did not include substrate- or microbe-only controls arm, so synergism could not be determined.

## Formulating Synergistic Synbiotics

Compared to complementary synbiotics, it is much more challenging to formulate and test synergistic synbiotics. As noted above, there are few reports in the literature that provide any specific rationale or strategy for how these synbiotics could be formulated (Kearney and Gibbons, 2018; Kumari et al., 2021). Without such a strategy, one cannot expect that combining a probiotic with a prebiotic will necessarily result in synergism. Rather, there should be a metabolic, functional, or other scientific basis for expecting the two components to provide a synergistic effect. Moreover, successful formulations of synergistic synbiotics requires an appreciation of the ecological constraints that exist in the gut environment. This is in contrast with the oversimplified notion that a single nutrient can drive colonization of a companion microbe (Edwards et al., 2020). This is especially apparent as recent research has substantiated the ecological complexity and individuality of the human gut microbiome (Nayak and Turnbaugh, 2016; Ryu et al., 2021) and the important role of fiber cross-feeding in the gut (Boger et al., 2018; Cantu-Jungles and Hamaker, 2020).

Indeed, it is conceivable that a prebiotic substrate could be partially degraded initially by members of the resident microbiota, with the remaining constituents consumed by and enriching the co-administered probiotic microbe, which then provides a health benefit on the host. Conversely, it is also possible that the added microbe converts the substrate into components that are then metabolized by commensal or companion microbes. An example of the latter case was described in an *in vitro* study (Boger et al., 2018), where investigators showed that a probiotic strain of *Lacticaseibacillus paracasei* subsp. *paracasei* (formerly *Lactobacillus)* produced a GH32 extracellular exo-inulinase that degraded fructans with a high degree of polymerization (DP). Smaller DP molecules were then formed which were consumed by companion lactobacilli unable to use larger DP substrates. Thus, these strains, combined with inulin or other longer chain fructans, could be formulated as cross-feeding synbiotic mixtures. A clinical trial would be necessary, however, to determine if this formulation provided a synergistic health benefit for the host.

Another example of synergism based on an indirect effect was recently reported for an oral synbiotic (Bijle et al., 2020). In this *in vitro* study, the well-studied probiotic, *Lacticaseibacillus rhamnosus* GG (formerly *Lactobacillus rhamnosus*), was paired with the amino acid, arginine. The goal was to develop a synbiotic that could inhibit *Streptococus mutans*, the causative agent of dental caries. Arginine is utilized by commensal microbes *via* the arginine deiminase system (ADS), yielding ammonia. The latter raises the pH, which is inhibitory to *S. mutans.* Although *L. rhamnosus* GG did not appear to express the ADS, its growth was enhanced by arginine, and it therefore indirectly contributed to *S. mutans* inhibition.

Polyphenol-based synbiotics may also be mediated by indirect metabolism in the gut, as recently proposed Sharma and Padwad (2020). Polyphenolic-rich foods, including red wine (Moreno-Indias et al., 2016), olive oil (Martín-Peláez et al., 2016), pomegranates (Cortés-Martín et al., 2021), and berries (Jamar et al., 2020) have been suggested to enrich for bifidobacteria. How their metabolism occurs in the gut is less clear, but appears to occur *via* several cross-feeding steps (Parkar et al., 2013). Thus, the polyphenolic substrate could be transformed directly into bioactive molecules that confer a health benefit on the host or indirectly into metabolites used by other microbes to produce beneficial end-products.

## Synergistic Statistics

Importantly, a clinical study must be conducted that is sufficiently powered such that it is possible to demonstrate that the effect of the synbiotic is statistically greater than the individual components, as well as the placebo. Several statistical tests are commonly used in pharmacology and biomedicine to determine synergism from biological datasets (Demidenko and Miller, 2019; Lederer et al., 2019), but are rarely applied to test for efficacy of synbiotics. These synergistic models are built on previous principles of non-interactions whereby interaction effects can be described as either synergistic or antagonistic. Similarly, these principles and statistical models can be used to determine significant synergistic effects whereby the prebiotic and probiotic act as independent references in a null-response model.

## Recent Approaches for Designing Synergistic Synbiotics

**FIGURE 1:**
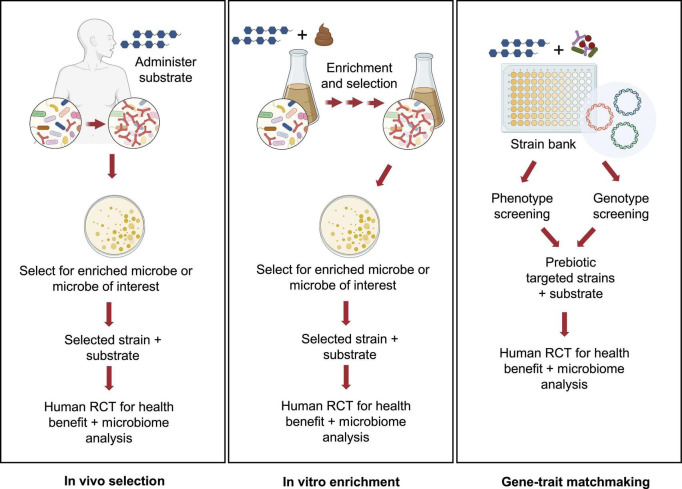
Pre-clinical approaches for identifying potential synergistic synbiotics. Several strategies for rationale identification of probiotic strains that can act synergistically with prebiotic substrates have been described in the literature. These pre-clinical approaches can be high-throughput and cost-effective implementations prior to a clinical study using either *in vivo* **(A)** or *in vitro* approaches **(B,C)**. Images created by BioRender.com.

Accounting for the considerable costs involved in conducting clinical trials, pre-clinical approaches to design and test for potential synergism may be warranted. Thus, identifying strains and substrates, *a priori*, that would be expected to satisfy criteria for synergistic synbiotics remains a formidable challenge. Toward this goal, several pre-clinical or *in vitro* platforms have been described (Figure 1).

This first approach, called *in vivo* selection (IVS), was described by Krumbeck et al. (2015). In an earlier human trial, GOS was administered to human subjects and GOS-enriched strains of bifidobacteria were isolated (Davis et al., 2010, 2011). Subsequent analyses revealed that one isolate, *Bifidobacterium adolescentis* IVS-1, had been enriched eightfold during GOS feeding in its autochthonous host. Pairing this strain with GOS provided a rational basis to test for synergism in a rodent model. Although IVS-1 alone enhanced relative abundance of *B. adolescentis* to about 3% in the colon, when combined with GOS, abundance increased to 37% and to more than 2 logs as measured by IVS-1 specific- qPCR. When the *B. adolescentis* IVS-1 strain (at 10^9^) was combined with GOS (5 g) in a human RCT, a treatment effect (improved gut barrier function) was observed compared to the baseline values (Krumbeck et al., 2018a). Although the IVS platform remains an ecologically rational approach to obtain putative synergistic synbiotics, its main disadvantage is that it requires human clinical trials from strain isolation to final efficacy studies.

An alternative approach, called *in vitro* enrichment (IVE), was described by Kok et al. (2019). The investigators used an *in vitro* method in which xylooligosaccharides (XOS) were added to fecal fermentation vessels, followed by daily stepwise dilutions to mimic gastrointestinal flux. Similar to the IVS approach, the IVE method has an ecological basis, in that the method selects for strains that were able to resist dilution pressure, grow rapidly, persist in the *in vitro* environment, and out-compete commensal microbes for the XOS substrate. After ∼20 generations, autochthonous strains enriched by XOS were isolated and identified. When one of the enriched strains, *Bifidobacterium longum* subsp. *longum* CR15, was re-inoculated into fresh fecal fermentations in the presence or absence of XOS, the CR15 strain persisted, but only when XOS was present. Thus, assuming enhanced persistence reflects synergism, the IVE platform would appear to be an effective high-throughput method for identifying putative synergistic synbiotic combinations prior to clinical trials.

Another *in vitro* approach was recently described that relied on matching phenotypic and genotypic properties of putative probiotic strains (Fuhren et al., 2020). A collection of 77 *Lactiplantibacillus plantarum* (formerly *Lactobacillus plantarum*) strains were screened for their ability to grow on a range of prebiotics, including GOS, FOS, inulin, isomaltooligosaccharides (IMO), arabinoxylooligosaccharides, and fucoidan along with characterization of carbohydrate utilization patterns identified through chromatographic methods. Genomes from strains having relevant substrate utilization phenotypes were analyzed to match those growth patterns to specific carbohydrate utilization gene clusters. Accordingly, strains were identified whose FOS, inulin, and IMO phenotypes were consistent with the presence of the relevant gene clusters. In a subsequent study, the researchers were able to identify genes involved in utilization of long chain GOS in strains of *L. plantarum*, suggesting that combining those strains with specific GOS fractions would enhance the selectivity of the formulated synbiotic (Fuhren et al., 2020). Ultimately, this high-throughput *in vitro* screening approach, like the IVE method, provides a rational basis for formulating highly selective synbiotic pairs prior to clinical trials.

## Recent Examples

The updated definition of a synergistic synbiotic does not require that the individual component parts (i.e., substrate and microbe) satisfy the probiotic or prebiotic definitions. This view was intended to provide a basis for innovative, ecologically based combinations of substrates and microbes. Accordingly, several examples have emerged in the past few years based on this understanding. One such example, consisting of *L. rhamnosus* GG and arginine, was described above (Bijle et al., 2020). Although neither of these individual components would appear to qualify, by definition, as oral probiotics or prebiotics, together they inhibited *S. mutans in vitro.* If this effect could be demonstrated, *in vivo*, it would meet the requirements of a synergistic synbiotic.

Another novel putative synbiotic formulation was recently described (Perraudeau et al., 2020) that contains *Bifidobacterium longum* subsp. *infantis*, plus four so-called next generation probiotic bacteria, including *Akkermansia muciniphila*, *Clostridium beijerincki*i, *Clostridium butyricum*, and *Anaerobutyricum hallii*. These strains were selected based on their ability to degrade oligosaccharides (*A. muciniphila* and *B. infantis*) and to produce butyrate (*A. hallii*, *C. beijerincki*, and *C. butyricum*). When combined with inulin and administered to participants with type 2 diabetes, the synbiotic improved postprandial glucose compared to a placebo treatment. However, this was a two-arm study, so it was not possible to ascribe the outcome to the synbiotic or to one of the individual components.

In addition to the complementary synbiotics described above for reducing sepsis and modulating microbiome composition in infants, other potentially synergistic synbiotics have also been developed using *B. infantis*. In a recent report (Barratt et al., 2022), infants with acute malnutrition were given *B. infantis* with or without HMO lacto-N-neotetraose. Although both treatments led to engraftment of the *B. infantis* strain and promoted weight gain in the infants, the synbiotic did not enhance the effect compared to the *B. infantis*-only treatment. This synergistic relationship between *B. infantis* and HMOs was also reported in Button et al. (2022) where *B. infantis*, which is typically absent in adults, could be engrafted in adults when paired with concentrated HMOs. In this study, the inclusion of the relevant controls (*B. infantis*-only and HMO-only) provided a basis for demonstrating specific utilization of the substrate by the microbe, one of the requirements for synergism.

Collectively, the studies described above suggest that an effective strategy for restoring bifidobacteria and repairing a dysfunctional microbiome in infants as well as adults is by supplementing feeding regimens with synbiotics containing selected strains and relevant substrates.

Finally, in addition to human studies, a synergistic synbiotic to promote animal health was recently investigated (Mohammadi et al., 2022). In this study, a lactic acid bacterium used as an animal feed supplement, Pediococcus *acidilactici* CNCM I-4622, was combined with a plant polysaccharide derived from pistachio nut hulls. Individually, these components enhanced growth and infectious disease resistance of farmed tilapia fish. However, when combined as a synbiotic, expression of immune biomarkers and resistance to *Aeromonas hydrophila*, a fish pathogen, were greater than the individual components, indicating the combination had synergistic activity. In addition, intestinal concentrations of acetic and propionic acids were higher in the synbiotic. The authors suggested these short chain fatty acids may contribute to the observed antibacterial activity as well as support growth of intestinal epithelial cells.

## Conclusion

Synbiotics have considerable potential to modulate the gut microbiome and improve human and animal health. From both a commercial, as well as a clinical perspective, enhanced health outcomes beyond that conferred by the probiotic microbe or the prebiotic substrate would be advantageous to consumers. Synergistic synbiotics can enhance establishment and/or persistence of the companion microbe and may improve clinical outcomes compared to probiotics or prebiotics alone. In addition, well-designed synergistic synbiotics can be an effective strategy to convert non-responders into responders, thereby increasing treatment efficacy in a greater number of consumers.

Despite these opportunities, synergistic synbiotics have been difficult to formulate and few, if any, have been shown to have clinical efficacy. Most clinical trials have not included relevant prebiotic or probiotic controls, and many have not conducted the relevant microbiota analyses. In addition, the rationale for selection of the prebiotic substrates and probiotic microbes is often absent. Certainly, formulation of effective prebiotics, probiotics, and synbiotics that can deliver health benefits will continue to rely on advances in gut microbiome research. In particular, the increased sequencing and computational capabilities will provide a better understanding of the complex ecological relationships within the gut microbiome and enhance development of synbiotics and other gut health products.

## Author Contributions

DGQ wrote the main draft and edited the manuscript. RH conceived, co-wrote, and edited the manuscript. CK co-wrote and edited the manuscript. All authors contributed to the article and approved the submitted version.

## Conflict of Interest

RH served on the Board of Directors for the International Scientific Association for Probiotics and Prebiotics and was a co-founder of Synbiotic Health, a manufacturer of probiotic microbes. The remaining authors declare that the research was conducted in the absence of any commercial or financial relationships that could be construed as a potential conflict of interest.

## Publisher’s Note

All claims expressed in this article are solely those of the authors and do not necessarily represent those of their affiliated organizations, or those of the publisher, the editors and the reviewers. Any product that may be evaluated in this article, or claim that may be made by its manufacturer, is not guaranteed or endorsed by the publisher.
